# Case study of using the single-atom R1 method to solve a small protein structure

**DOI:** 10.1107/S2053273326005668

**Published:** 2026-06-25

**Authors:** Xiaodong Zhang

**Affiliations:** a Chemistry Department Tulane University 6400 Freret Street New Orleans Louisiana70118 USA; Institute of Crystallography - CNR, Bari, Italy

**Keywords:** single-atom R1, solvent diffraction effects, padding additional C atoms, structure solution

## Abstract

A small protein structure would not yield a solution via the single-atom R1 (sR1) method. A salient sign of trouble was that many atoms were dubiously accepted into the model because their introduction caused the sR1 value to increase. A plausible hypothesis is that this was very likely due to a hidden solvent diffraction effect. After accounting for this hidden solvent effect via padding additional C atoms, this small protein structure now has been successfully solved by the sR1 method. Further analysis of the protein crystal density indicated that the above successful padding of C atoms had over-accounted for the solvent content. However, empirical observations have indicated that, when an initial application of the sR1 method shows that many atoms are not welcomed into the model, padding numerous C atoms (even if over-accounting for the solvent content) benefits the sR1 calculation by making spotting plausible fragments more reliable and/or making the resulting model more complete.

## Abstract 2

A small protein structure has been successfully solved by the single-atom R1 method after accounting for the hidden solvent diffraction effects.

## 1. Introduction

In X-ray crystallography, many programs/algorithms for solving crystal structures, for example, *SHELXT* (Sheldrick, 2015 ▸) and *SIR* (Burla *et al.*, 2015 ▸), and even large program packages like *CCP4* (Agirre *et al.*, 2023 ▸) *etc*., are based on phasing (Giacovazzo, 2014 ▸), which is typically taken care of by a dual-space recycling process, utilizing reciprocal-space phase refinement and/or real-space electron-density modifications (Weeks *et al.*, 1993 ▸; Miller *et al.*, 1993 ▸; DeTitta *et al.*, 1994 ▸). In comparison, the single-atom R1 (sR1) method for solving crystal structures (Zhang & Donahue, 2024 ▸) is particularly interesting because it does not involve phasing. Instead, it relies on optimization of an sR1 function to introduce the atoms into a model one atom at a time. Another method based on a diagonal least-squares technique (Burla *et al.*, 2018 ▸) has a similar capability of solving a structure without involving phasing, even though it was used to produce *ab initio* phasing for small crystal structures.

When testing the sR1 method on various datasets, a particular dataset of a small protein, the SI form crambin (a plant seed protein) (data taken from the Protein Data Bank code 1AB1; Yamano *et al.*, 1997 ▸), was very puzzling. This structure contains 658 atoms in its unit cell. Though other structures of similar sizes readily yielded to the sR1 method, this structure stubbornly refused to yield. Trials of the sR1 method on this dataset for nearly 2 years failed to produce a solution. Only recently has a plausible hypothesis been suggested, causing the mystery to be solved. This paper reports some analysis of the puzzle and a simple trick to overcome the difficulty.

## 2. A brief recap of the sR1 method

Details of the sR1 method were published previously (Zhang & Donahue, 2024 ▸). The main idea of this method is recapped here. Consider a crystal structure with *N* atoms in its unit cell. To start locating the atoms, the very first (the heaviest) atom can be assigned to an arbitrary position. To locate the second (the heaviest among the remaining) atom, an sR1 function can be defined by modifying the traditional R1 factor via removing its dependence on the position of atoms 3, 4, …, to *N*. This approximate R1 factor only depends on the location of atom 2, even though it does contain the position of atom 1 as a known parameter. Therefore, the location of atom 2 can be determined by globally minimizing this sR1 function. Proceeding in a similar manner, atoms 3, 4, …, to *N* can all be located one by one via minimizing the sR1 functions corresponding to each of these atoms in sequence. In this way, in one cycle of the sR1 calculation, a tentative full model is produced. However, a user needs to examine this resulting model and either select a plausible fragment or fragments, or delete ghost atoms, to get a partial model. Starting from this partial model, another sR1 cycle can be repeated to yield the next improved partial model. This step should be repeated until there is no more improvement. The final partial model is the solution reached by the method.

The sR1 method has been tested on more than approximately 200 datasets (a list is available from the corresponding author by request). For most of these cases, the calculation is straightforward because the very first cycle reveals chemically meaningful partial structures. There are troublesome cases where barely any plausible fragments can be spotted in the first tentative full model. In such cases, a user must pick a plausible fragment or fragments based on their best intuition. If the guess is close to correct, more plausible fragments appear in the new result and can be selected to start the further cycles, and eventually the calculation converges to the correct solution. Note that, by the nature of starting the very first atom with a randomly generated position, and by the nature of a user interfering subjectively, for the same dataset, each trial of the method reaches the solution via a very different path. However, it has been observed that these paths all converge on the same solution, as judged by the fact that these solutions can overlap atom by atom within about 0.5 Å after inverting and/or shifting one of them.

## 3. A plausible hypothesis: the hidden solvent diffraction effects

When applying the sR1 method to solve the crambin structure, a salient sign of trouble was that in one cycle of the sR1 calculation many atoms (as many as 337 atoms) were not welcomed into the model; that is, their introduction into the model caused the sR1 value to increase. This sign was probably due to not accounting for the hidden solvent diffraction effects in the calculation. To account for the solvent diffraction effects, the solvent atoms need to be added to the cell content. Once added to the cell content, even though the calculation does not seek to locate these atoms, the baseline effects of these atoms will be included in the calculation. Typically, solvents comprise light atoms, so an easy remedy is to pad additional C atoms into the content. A few calculations were performed by starting from a single S atom and running one sR1 calculation cycle for a few trial padding C atom numbers. It was observed that, without padding any C atoms, there were 337 atoms that caused the sR1 to increase. This number dropped to 54 if 396 C atoms were padded, to 1 if 658 C atoms were padded, and finally to 0 if 796 C atoms were padded. To sufficiently account for the effect, it was decided to pad 796 C atoms, which happened to lead to a success (see the next section). Note that the number 658 was the total number of atoms in the target structure, and the numbers 396 and 796 were selected because they resulted in changing the total C atoms in the cell from 404 to 800 and 1200, respectively. So, the selections were made roughly, and were not as precise as they appeared to be. It appeared that among these four tried padding numbers, padding more was better (see the *Discussion* section below).

## 4. Solving the crambin structure after padding 796 C atoms

At the start, the sR1 program generated a random position for the first S atom. Next, starting from this single S atom after one sR1 calculation cycle, the first tentative full model was generated. However, there was no indication of chemically meaningful organization. From this result, a few atoms were picked, based on best intuition (namely, the atoms formed a plausible fragment or fragments), for starting the second calculation cycle. Such a process was repeated for a few cycles. Gradually, more chemically plausible organization appeared, as judged by the bond lengths and angles. Thus, gradually, more fragments could be picked for starting the next cycle. Then, at the end of a particular cycle, suddenly, a chemically meaningful structure appeared. From that point on, instead of small fragments being picked, ghost atoms were deleted to form the starting model for the next calculation cycle. This step was repeated until there was no more improvement. Some details of this calculation have been included in the supporting information.

## 5. Comparing the resulting model with the correct model

The resulting model had 581 atoms. Compared with 658 atoms in the correct model, the resulting model was 77 atoms fewer. The resulting model could be shifted such that its 12 S atoms were optimally matching those in the correct model. After shifting, each atom in the resulting model matched one corresponding atom in the correct model. The distances between the two atoms of all corresponding pairs were calculated. Among 581 pairs, 563 pairs had distances between 0 and 0.2 Å. Only 18 pairs had distances between 0.2 and 0.41 Å. As a result, the calculation had yielded a model of high quality. (Note that this partial model was further improved to a model containing all 658 atoms by bond length guided sR1 calculations. Some details of this are given in the supporting information.)

## 6. A working hypothesis

The exact mathematical mechanism of how padding additional C atoms means that more atoms can be introduced into the model without causing the sR1 value to increase is not immediately obvious. However, intuition points to the idea that when more and more atoms are introduced into the model, eventually new introductions will cause the sR1 to increase; therefore, if more atoms are assumed to exist in the cell, the increase in the sR1 will be delayed, and it will be delayed beyond the expected cell content if numerous C atoms are padded into the cell. However, whether padding benefits the sR1 calculation or not can only be determined by empirical observations (see details in the next section).

## 7. Discussion

One suspects that padding 796 C atoms overestimates the solvent content in the unit cell. This suspicion is confirmed by calculating the crystal mass density. Table 1 ▸ shows the calculated mass density at each tried padding number. It is widely accepted that a dry protein has mass density about 1.35 g cm^−3^ (Andersson & Hovmöller, 1998 ▸). Without padding C atoms, the calculated crystal mass density is 0.88 g cm^−3^. This means the protein occupies 0.88/1.35 = 65% of the unit-cell volume. Assuming the rest of the 35% cell volume is occupied by water (of density 1 g cm^−3^), the crystal mass density is estimated to be 1.22 g cm^−3^. According to this estimation, all three tried padding numbers result in overestimating the solvent content; in particular, when padding 796 C atoms the calculated crystal mass density is 1.83 g cm^−3^, which is much higher than 1.22 g cm^−3^.

However, padding any number of C atoms is always mathematically possible. There is no need to worry whether this is physically feasible or not. The only concern should be: does over-accounting for the solvent content harm or benefit the sR1 calculation? It was known that the sR1 method can always reach a good partial model of the crambin structure (even without padding any C atoms) if the starting model is the 12 correctly positioned S atoms. So, the above question can be answered by performing one cycle of such sR1 calculation for each of the four padding numbers and comparing the resulting tentative full models with the correct model. The results of such comparison are also shown in Table 1 ▸. It is seen that without padding C atoms the resulting model has 567 atoms located within 0.6 Å. When padding 396 C atoms this number is improved to 569 and further improved to 576 and 577 if padding 658 and 796 C atoms, respectively. Thus, for the crambin case, padding C atoms helps the sR1 method to reach a more complete model, and it appears padding more is better, even though all these padding numbers are overestimating the solvent content.

The number of dubiously accepted atoms – those when introduced into the model cause the sR1 to increase – is also listed in Table 1 ▸. Without padding any C atoms there are 127 atoms that are dubiously accepted into the model. For all three padding cases this number drops to 0. These results are observed when the starting model is the correctly positioned 12 S atoms. If a single S atom is used as the starting model (such calculations have been given in Section 2), there are 337 dubiously accepted atoms if no C atoms are padded, and this number drops to 54 if 396 C atoms are padded, to 1 if 658 C atoms are padded, and finally to 0 if 796 C atoms are padded. The existence of dubiously accepted atoms is an indication that the real signal is losing a battle against the noise, resulting in the atoms being positioned in random locations. This makes the starting sR1 cycles difficult to handle if it happens that those cycles do not yield an obviously chemically meaningful partial structure – because then hardly any plausible fragments can be spotted for starting the next sR1 cycle. Padding C atoms to prevent dubiously accepted atoms helps to overcome this difficulty as shown by the fact that without padding C atoms the crambin structure was not solved in numerous sR1 efforts for nearly 2 years, and once 796 C atoms were padded the structure was solved in a few weeks.

In retrospect, the author also recalled a few other cases in which many atoms were not welcomed into the models. Though these cases yielded to the sR1 method without padding C atoms, revisiting them with the new trick of padding additional C atoms has indicated that this approach is also effective in these cases, in terms of making the calculations smoother and the resulting models more complete. Details of one such example are included in the supporting information.

Out of curiosity, a couple of cases where there was no sign of trouble of any unwelcomed atoms were tested by intentionally padding as many C atoms as the total number of atoms in the target structure. It was observed that, in these cases, padding C atoms made the method perform slightly less effectively but otherwise the method still worked. Details of one such case have been included in the supporting information. Such study indicates that padding C atoms is not advised for general use, even though such general application only hurts the sR1 method slightly.

## 8. Conclusion

If an initial application of the sR1 method shows that many atoms are not welcomed into the model – namely, their introduction into the model causes the sR1 value to increase – a good trick to try is to add numerous (about the order of the total number of atoms of the target model) additional C atoms to the cell content. This trick makes the identification of plausible fragments more reliable for starting the next sR1 calculation cycles. It also helps the sR1 method to reach a more complete model.

## 9. Computer and software used

A Microsoft Surface Pro 9 [12th-generation Intel Core i7-1255U (2.60 GHz), 32.0 GB installed RAM] was used. The time taken by one sR1 cycle for solving the crambin structure ranged from 2 to 12 h, depending on how complete the starting model was. Software for the sR1 method was coded in Python. The Python codes are available on GitHub: https://github.com/xzhang222/sR1-method. A text file README.md explains how to use the Python codes to peform the sR1 calculation.

## Supplementary Material

Supporting information. DOI: 10.1107/S2053273326005668/ae5182sup1.pdf

## Figures and Tables

**Table 1:** Comparison of different C atom padding numbers

C atom padding number	Crystal density (g cm^−3^)	No. of atoms located within 0.6 Å†	No. of atoms dubiously accepted†
0	0.88	567	127
396	1.36	569	0
658	1.67	576	0
796	1.83	577	0

† These results were obtained from one sR1 cycle starting from 12 correctly positioned S atoms and comparing the resulting model with the correct model.

## References

[bb1] Agirre, J., Atanasova, M., Bagdonas, H., Ballard, C. B., Baslé, A., Beilsten-Edmands, J., Borges, R. J., Brown, D. G., Burgos-Mármol, J. J., Berrisford, J. M., Bond, P. S., Caballero, I., Catapano, L., Chojnowski, G., Cook, A. G., Cowtan, K. D., Croll, T. I., Debreczeni, J. É., Devenish, N. E., Dodson, E. J., Drevon, T. R., Emsley, P., Evans, G., Evans, P. R., Fando, M., Foadi, J., Fuentes-Montero, L., Garman, E. F., Gerstel, M., Gildea, R. J., Hatti, K., Hekkelman, M. L., Heuser, P., Hoh, S. W., Hough, M. A., Jenkins, H. T., Jiménez, E., Joosten, R. P., Keegan, R. M., Keep, N., Krissinel, E. B., Kolenko, P., Kovalevskiy, O., Lamzin, V. S., Lawson, D. M., Lebedev, A. A., Leslie, A. G. W., Lohkamp, B., Long, F., Malý, M., McCoy, A. J., McNicholas, S. J., Medina, A., Millán, C., Murray, J. W., Murshudov, G. N., Nicholls, R. A., Noble, M. E. M., Oeffner, R., Pannu, N. S., Parkhurst, J. M., Pearce, N., Pereira, J., Perrakis, A., Powell, H. R., Read, R. J., Rigden, D. J., Rochira, W., Sammito, M., Sánchez Rodríguez, F., Sheldrick, G. M., Shelley, K. L., Simkovic, F., Simpkin, A. J., Skubak, P., Sobolev, E., Steiner, R. A., Stevenson, K., Tews, I., Thomas, J. M. H., Thorn, A., Valls, J. T., Uski, V., Usón, I., Vagin, A., Velankar, S., Vollmar, M., Walden, H., Waterman, D., Wilson, K. S., Winn, M. D., Winter, G., Wojdyr, M. & Yamashita, K. (2023). *Acta Cryst.* D**79**, 449–461.

[bb2] Andersson, K. M. & Hovmöller, S. (1998). *Z. Kristallogr. New Cryst. Struct.* **213**, 369–373.

[bb3] Burla, M. C., Caliandro, R., Carrozzini, B., Cascarano, G. L., Cuocci, C., Giacovazzo, C., Mallamo, M., Mazzone, A. & Polidori, G. (2015). *J. Appl. Cryst.* **48**, 306–309.

[bb4] Burla, M. C., Carrozzini, B., Cascarano, G. L., Giacovazzo, C. & Polidori, G. (2018). *Acta Cryst.* A**74**, 123–130. 10.1107/S2053273318001407 29493541

[bb5] DeTitta, G. T., Weeks, C. M., Thuman, P., Miller, R. & Hauptman, H. A. (1994). *Acta Cryst.* A**50**, 203–210. 10.1107/s0108767393008980 8166951

[bb6] Giacovazzo, C. (2014). *Phasing in Crystallography – a Modern Perspective.* Oxford University Press.

[bb7] Miller, R., DeTitta, G. T., Jones, R., Langs, D. A., Weeks, C. M. & Hauptman, H. A. (1993). *Science* **259**, 1430–1433. 10.1126/science.8451639 8451639

[bb8] Sheldrick, G. M. (2015). *Acta Cryst.* A**71**, 3–8.

[bb9] Weeks, C. M., DeTitta, G. T., Miller, R. & Hauptman, H. A. (1993). *Acta Cryst.* D**49**, 179–181. 10.1107/S090744499200876X 15299558

[bb10] Yamano, A., Heo, N. H. & Teeter, M. M. (1997). *J. Biol. Chem.* **272**, 9597–9600. 10.1074/jbc.272.15.9597 9092482

[bb11] Zhang, X. & Donahue, J. P. (2024). *Acta Cryst.* A**80**, 237–248. 10.1107/S2053273324001554 PMC11067948 38497453

