# Supplementary material for: Case study of using the single-atom R1 method to solve a small protein structure
Source: Acta Crystallogr A Found Adv. 2026 Jun 25;82(Pt 4):331–4. doi: 10.1107/S2053273326005668 (PMC13325186; doi:10.1107/S2053273326005668)
Supplement: Supplementary file 1 [file a-82-00331-sup1.pdf]

Acta Cryst A FOUNDATIONS ADVANCES

Volume 82 (2026)

Supporting information for article:

Case study of using the single-atom R1 method to solve a small protein structure

Xiaodong Zhang

S1. The sR1 calculation cycles in solving the crambin structure

Cycle #1:

The program generated a random position for a single S atom, then expanded to a tentative full model:

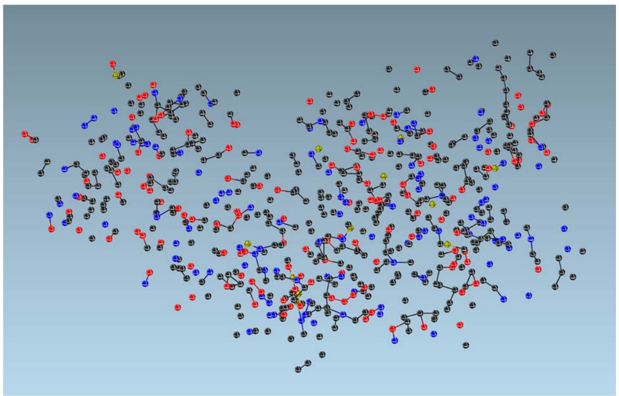

Cycle #2:

A few atoms were picked based on best intuition (namely, forming a plausible fragment):

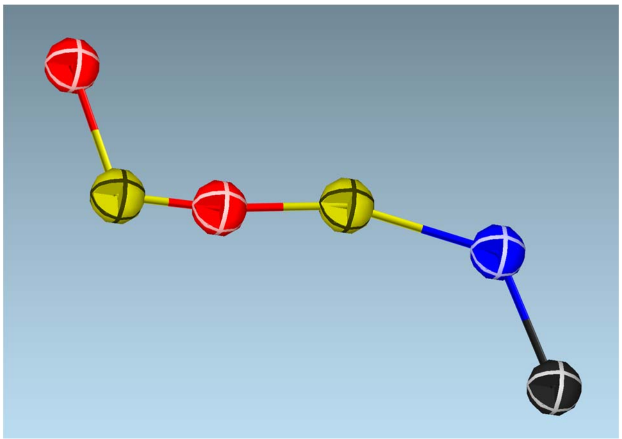

Then expand to the next tentative full model:

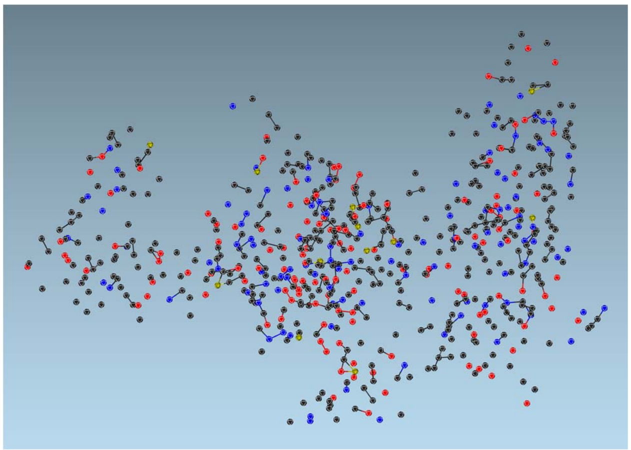

Cycle #3:

Now pick two small pieces:

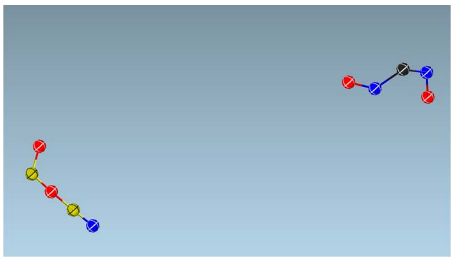

Expand to the next tentative full model:

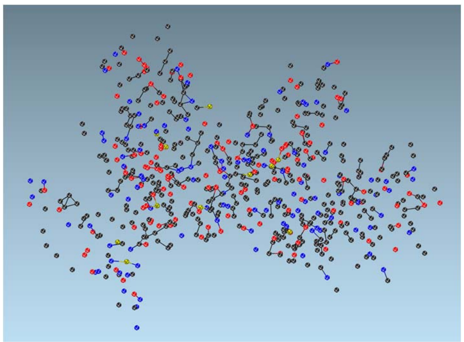

Cycle #4:

Now pick three small pieces:

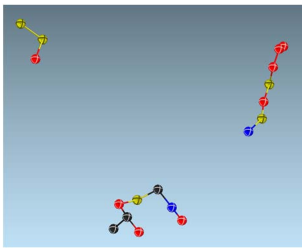

And then expand to the next tentative full model:

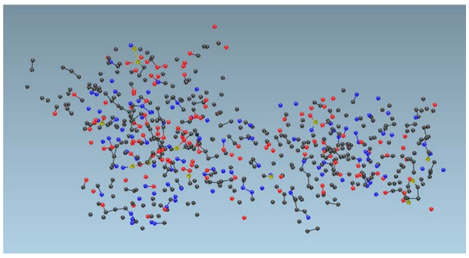

Cycle #5:

Now pick four small pieces:

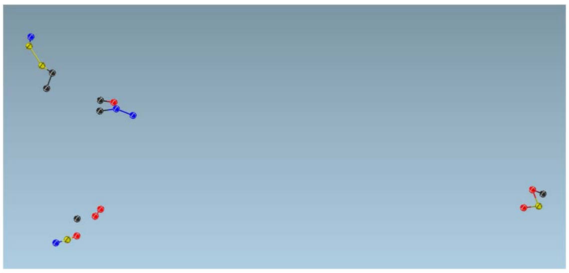

And expand to the next tentative full model:

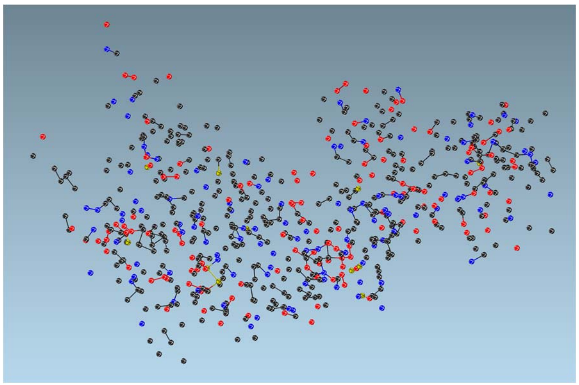

Cycle #6:

This time only pick three small pieces, but include more S atoms, and make cleaner choice:

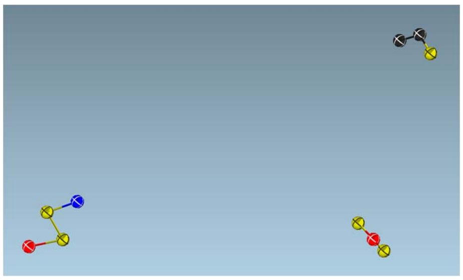

And again, expand to the next tentative full model:

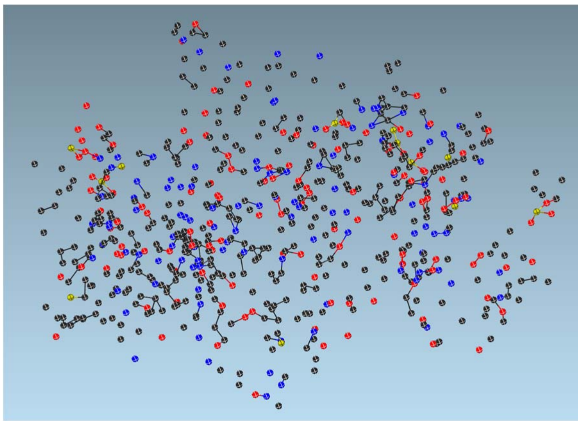

Cycle #7:

Pick five small pieces:

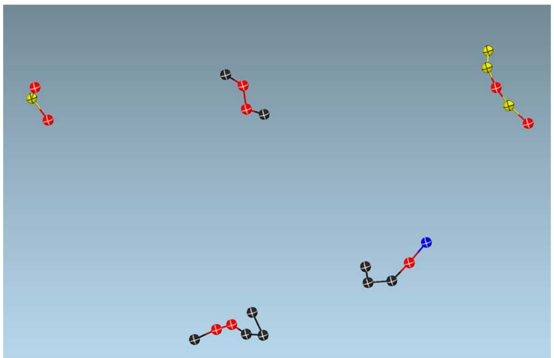

And expand to the next tentative full model:

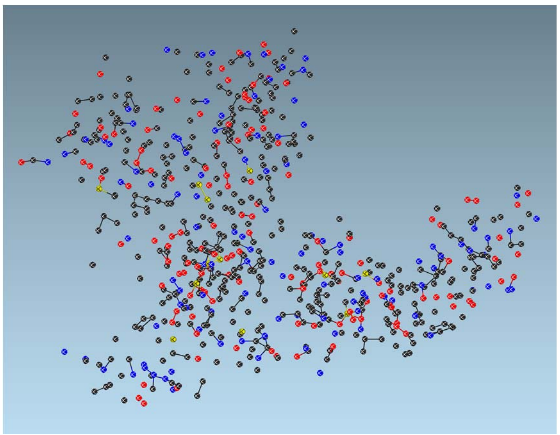

Cycle #8:

Pick 8 pieces:

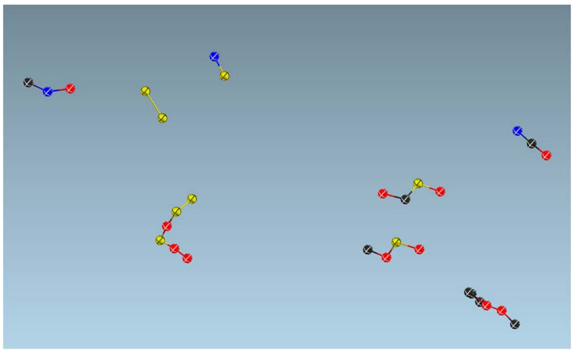

And expand to the next tentative full model:

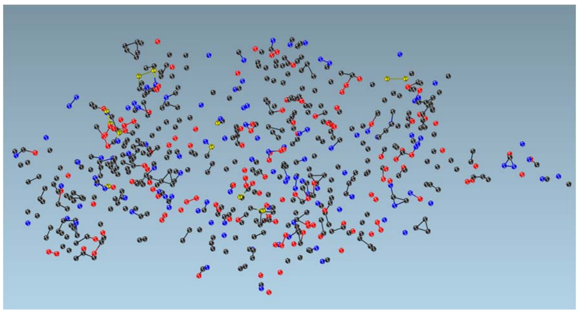

Cycle #9:

Pick 6 pieces including three S-S dimers:

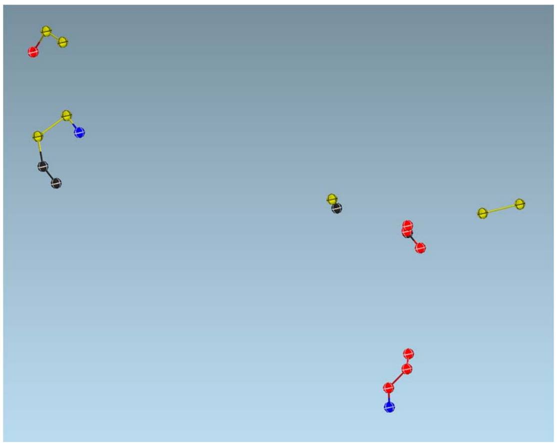

And expand to the next tentative full model:

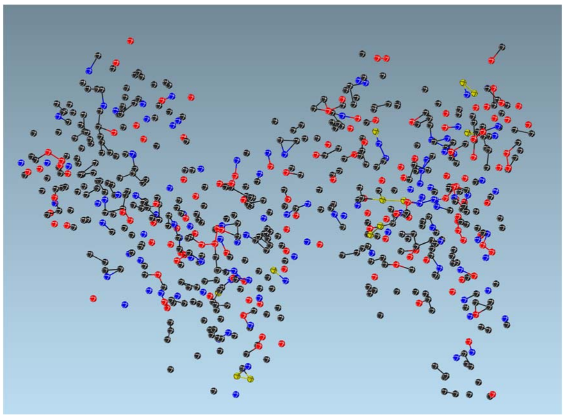

Cycle #10:

Pick 8 pieces including three S-S dimers:

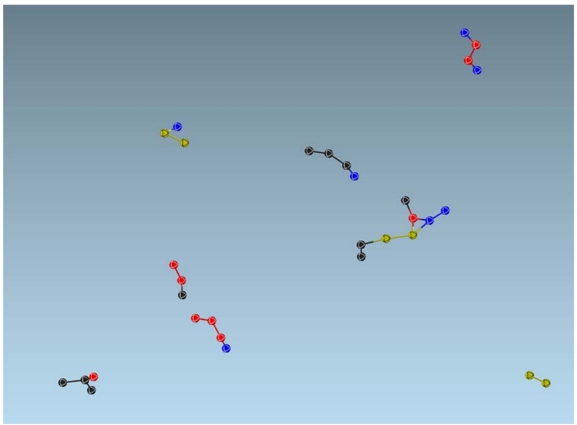

And expand to the next tentative full model:

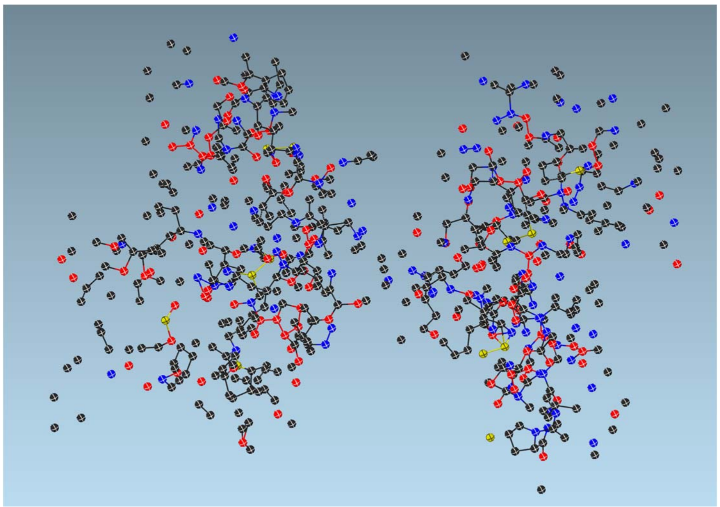

Cycle #11:

Suddenly, chemically meaningful structures have appeared. Delete the ghost atoms to get the following partial model:

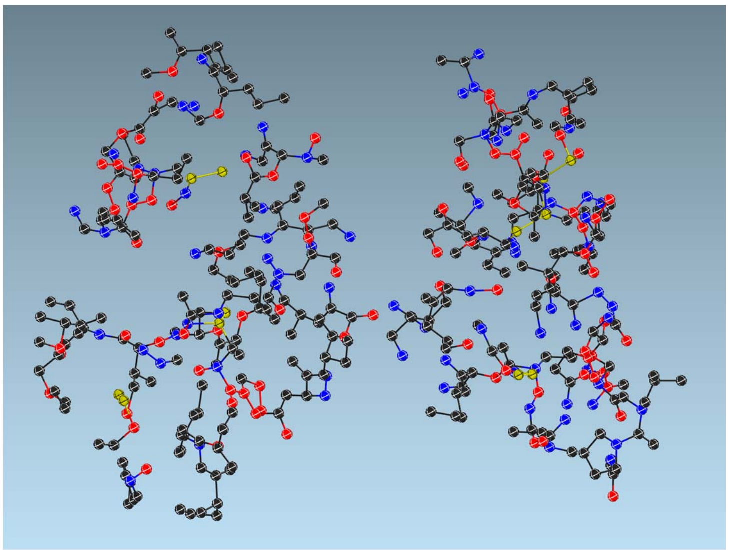

And expand to the next tentative full model:

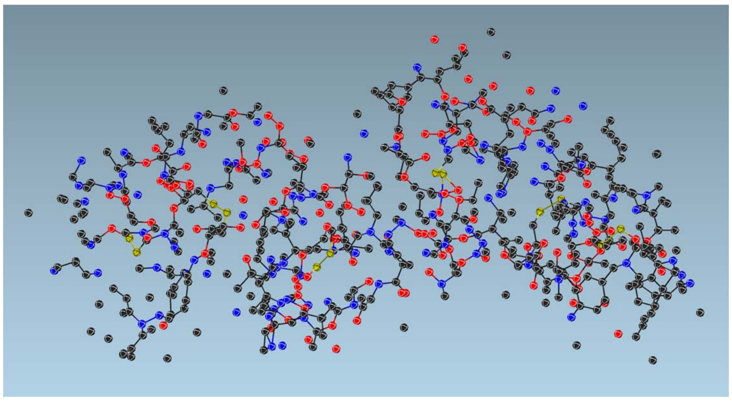

Cycle #12:

Delete the ghost atoms to get the next partial model:

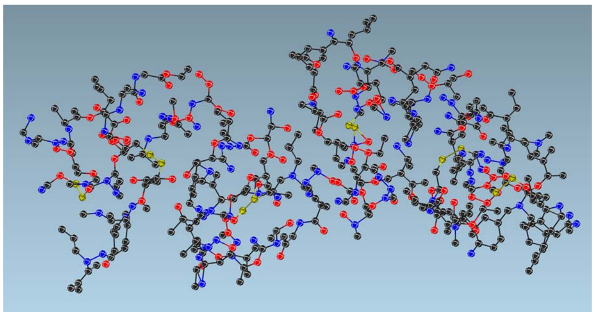

Then expand to the next tentative full model:

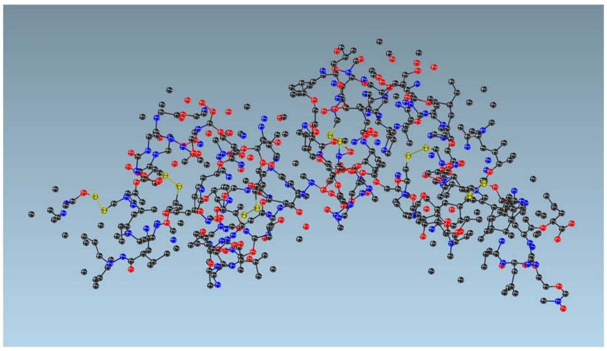

Cycle #13:

Delete the ghost atoms to get the next partial model:

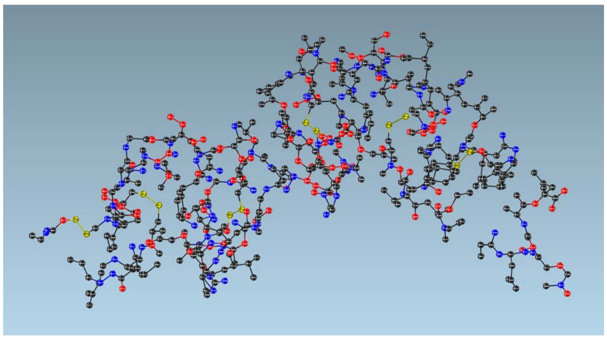

And expand to the next tentative full model:

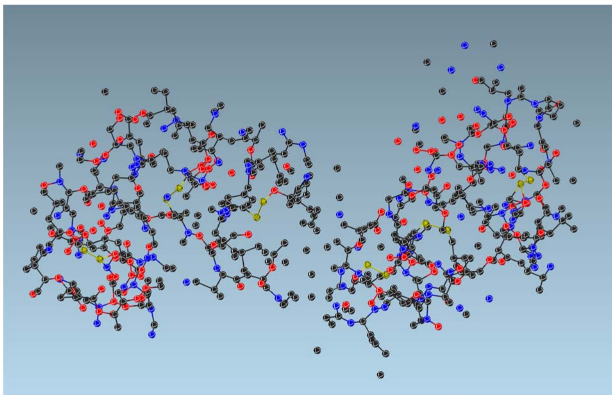

Cycle #14:

Delete the ghost atoms to get the next partial model:

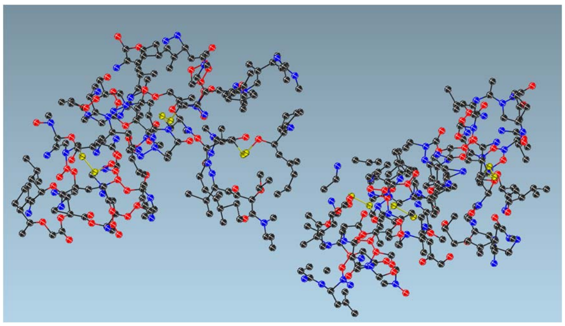

And expand to the next tentative full model:

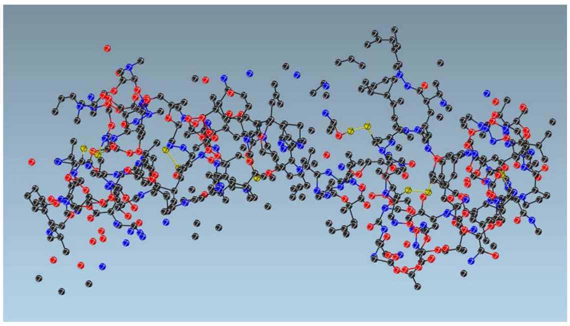

Cycle #15:

Delete the ghost atoms to get the next partial model:

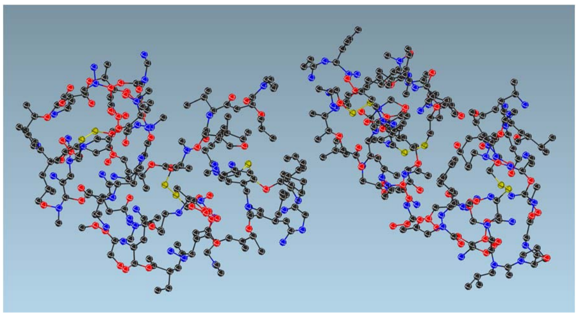

And expand to the next tentative full model:

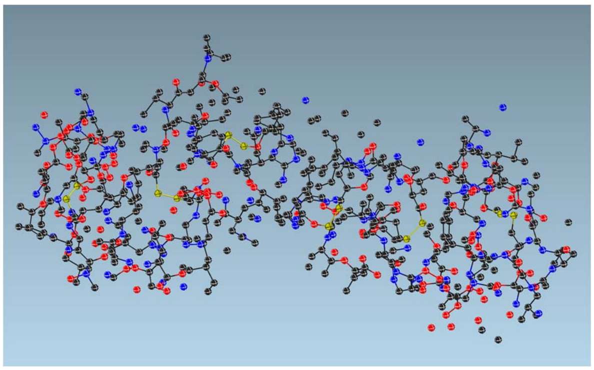

Cycle #16:

Delete the ghost atoms to get the next partial model:

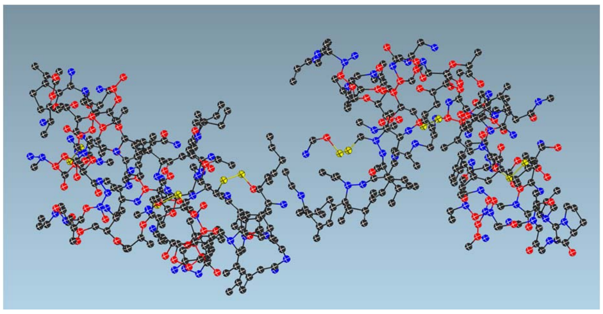

And expand to the next tentative full model:

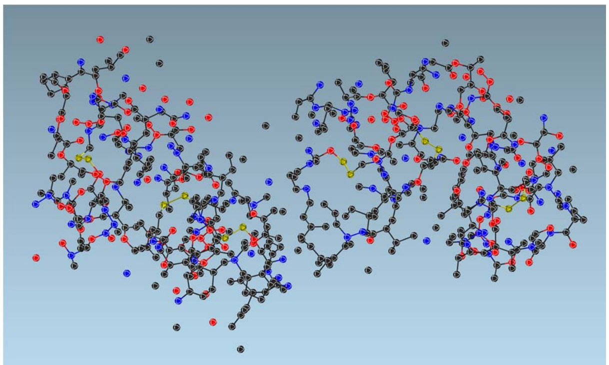

Cycle #17:

Delete the ghost atoms to get the next partial model:

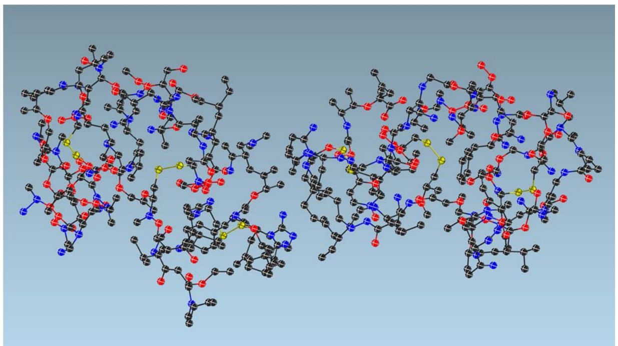

And expand to the next tentative full model:

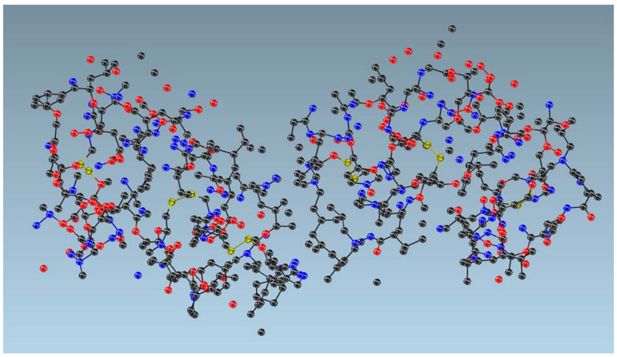

Final step:

Delete the ghost atoms to get the next partial model:

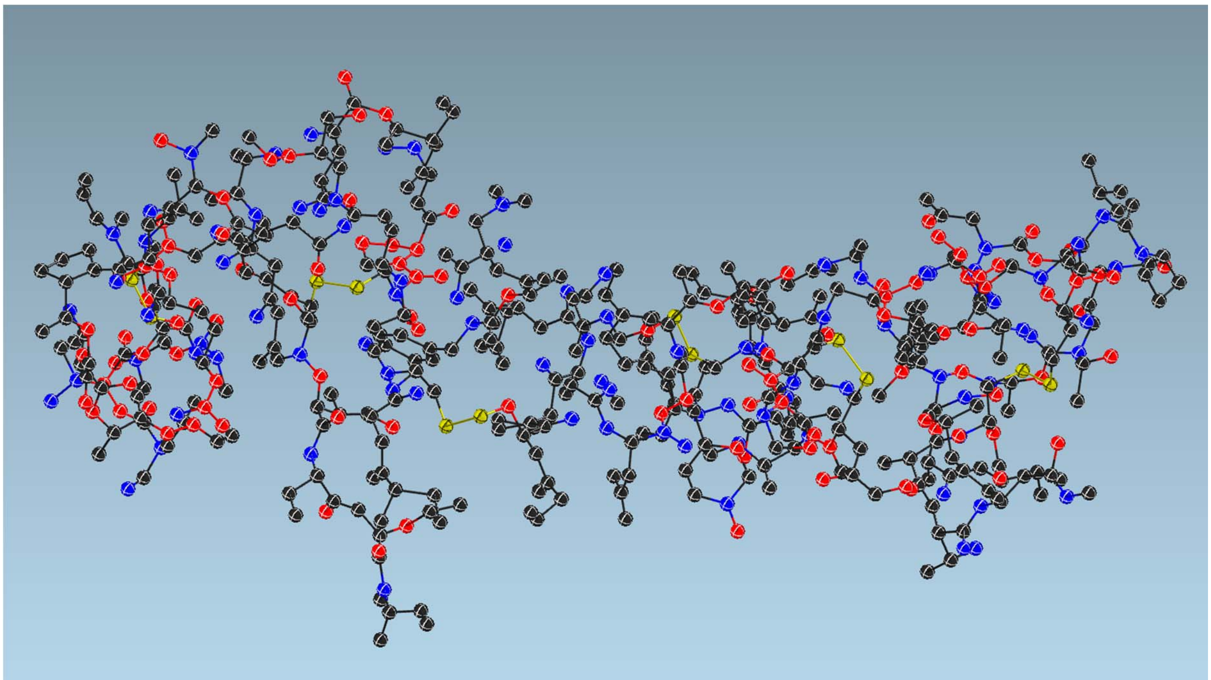

As there has been no more improvement, this is the final calculated model.

For comparison, the following is the correct model:

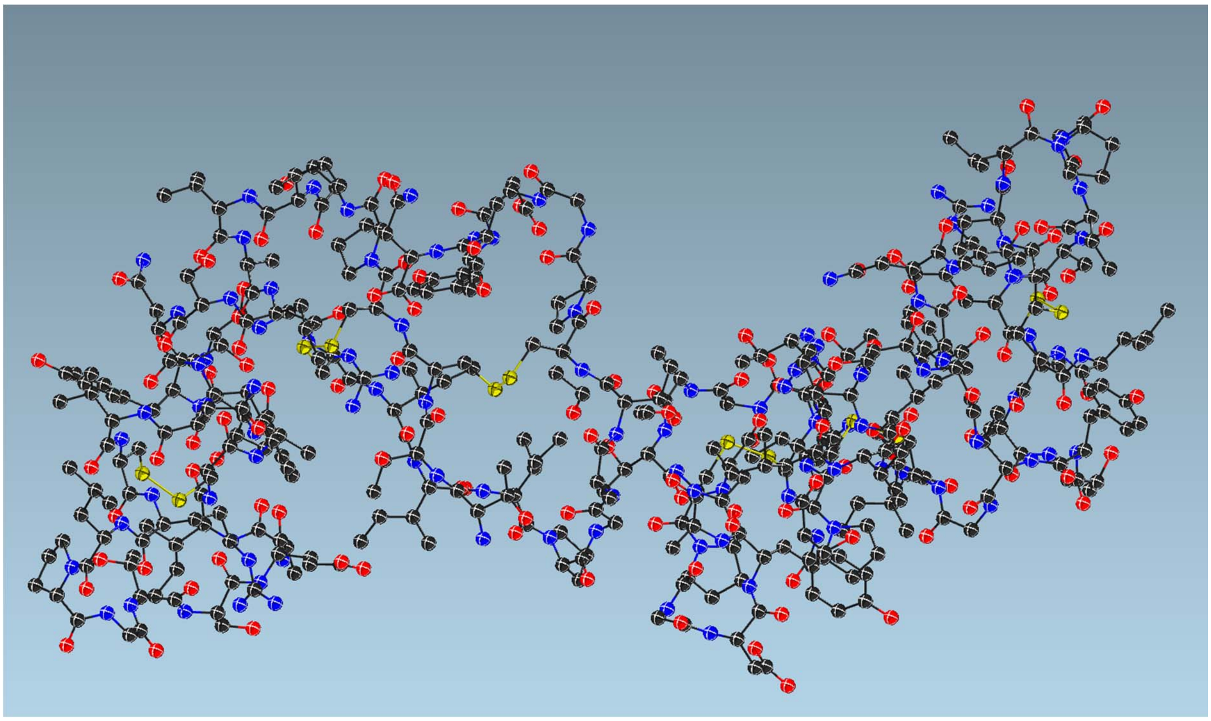

S2. Using bond length guided sR1 calculations to complete the crambin model

The crambin model can be further completed by a simple sR1 technique. This technique should be guided by the known amino sequence. To reduce efforts, here, the known correct model is used as guidance. By comparing the partial model with the correct model, a missing atom can be identified, call it as atom A. Further, the atom to which this missing atom is attached can be identified; call it as atom M. To locate the missing atom A, the sR1 search range can be limited within a spherical shell, which is centered on atom M, with bond length of M-A within a range, specifically here this range is 1.09 to 1.69 Å. Using this technique, the model can be completed. The following statistics show how the resulting model compares with the correct model:

601 atoms located within 0 to 0.2 Å

35 atoms located within 0.2 to 0.4 Å

6 atoms located within 0.4 to 0.6 Å

5 atoms located within 0.6 to 0.8 Å

3 atoms located within 0.8 to 1.0 Å

8 atoms located within 1.2 Å to infinite

There are 8 atoms that are obviously incorrectly located. The resulting model is shown below with these 8 atoms displayed as larger balls: (note that the atom types have been edited)

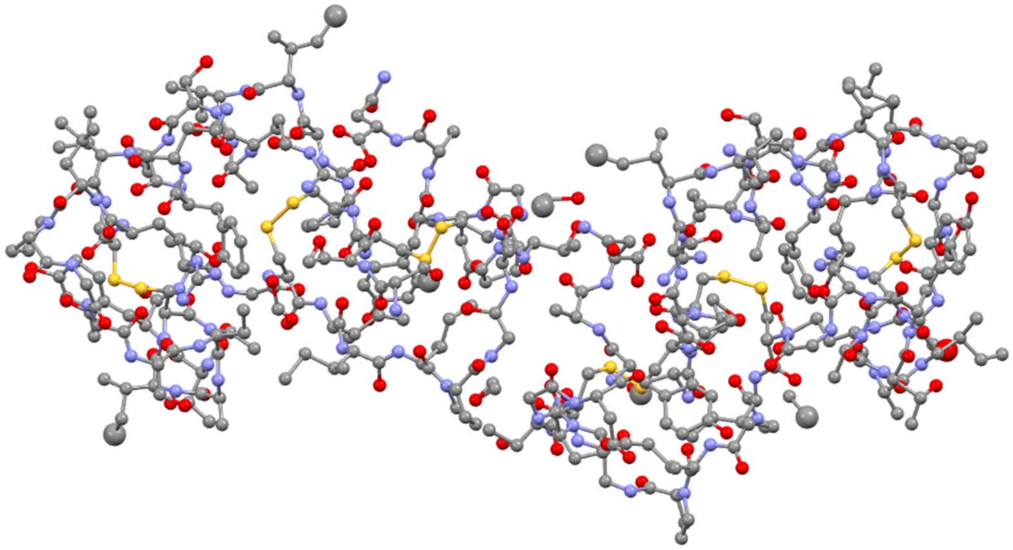

S3. Another example where the initial application of sR1 method shows dubiously accepted atoms and padding C atoms helps the sR1 calculation

Dataset code: JPD1253.

In this example, there are 86 atoms in the unit cell. The cell content is represented by CuS2P2F12N8C18 and Z = 2. The space group is P2(1)2(1)2.

Without padding any C atoms, starting from a single Cu atom, in one sR1 cycle, there are 7 dubiously accepted atoms, and the resulting model contains 66 atoms:

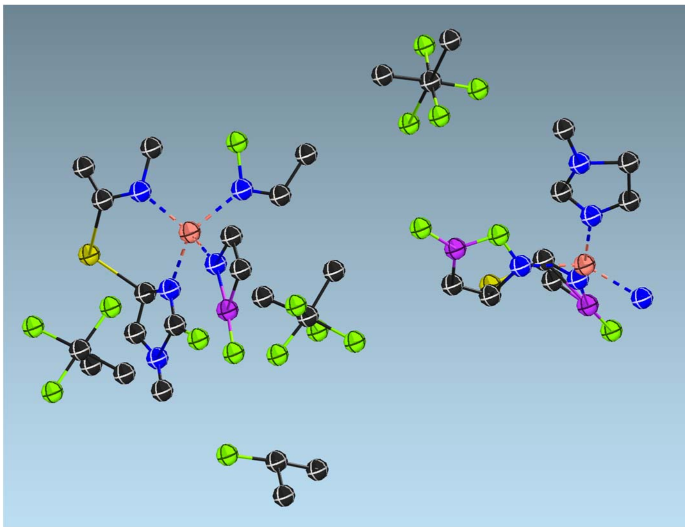

After additional four cycles, in which cycle 4 offers no improvement over cycle 3, the final model has 76 atoms:

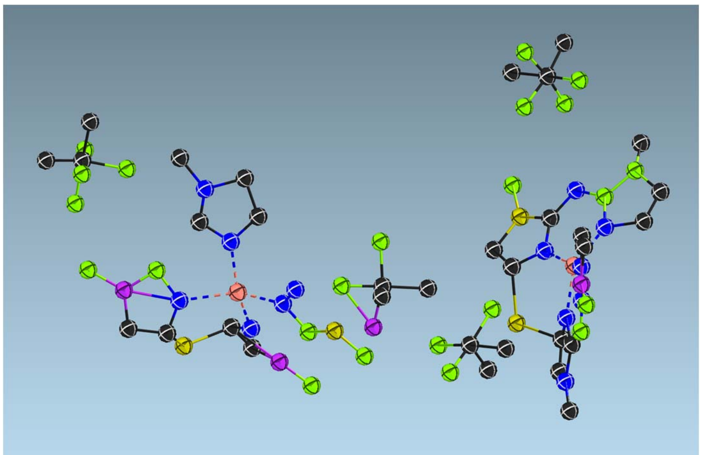

For comparison, the correct model has 86 atoms:

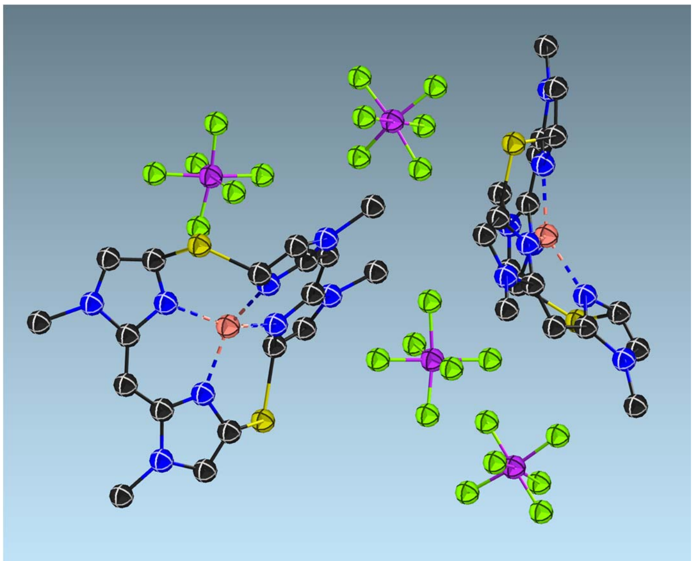

If padding 64 C atoms, starting from a single Cu atom, in one sR1 cycle, the number of dubiously accepted atoms is 0, and the resulting model has 73 atoms:

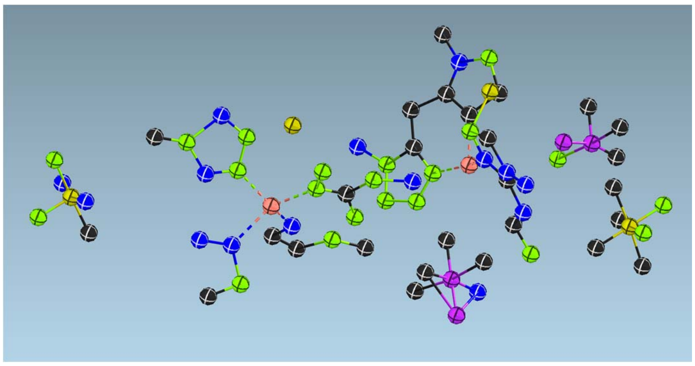

After additional four cycles, in which cycle 4 offers no improvement over cycle 3, the final model has 83 atoms:

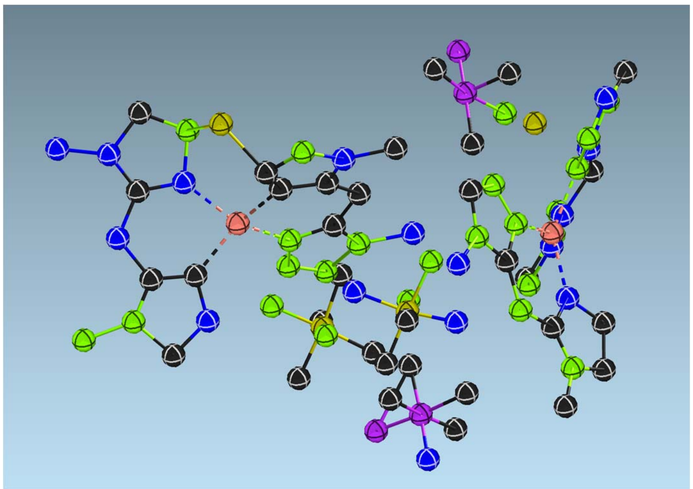

Conclusion: padding 64 C atoms helps the sR1 method to reach a more complete model.

S4. One example where the initial application of sR1 method does not show any dubiously accepted atoms and padding C atoms hurts the sR1 calculation slightly

Dataset code: JPD1249.

In this example, there are 32 atoms in the unit cell. The cell content is represented by S2O2C12 and Z = 2. The space group is P2(1).

Without padding any C atoms, starting from a single S atom, in one sR1 cycle, there are 0 dubiously accepted atoms, and the resulting model contains 32 atoms:

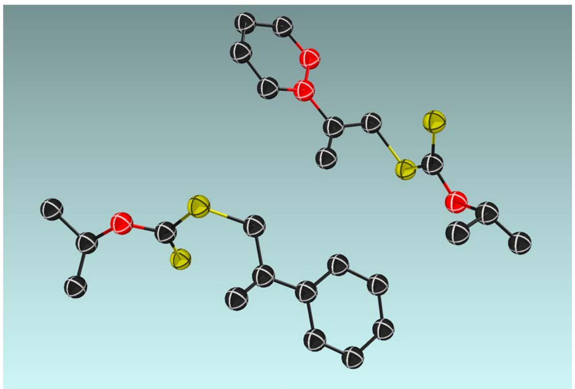

This matches the correct model:

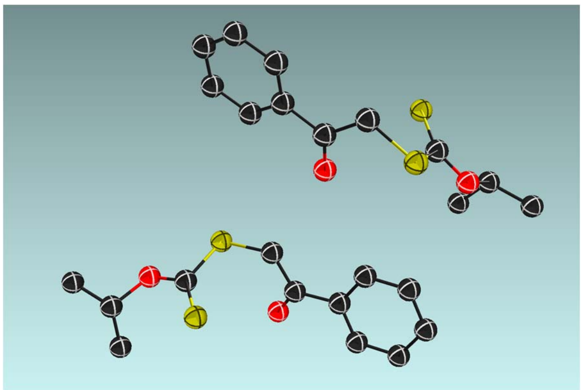

If padding 32 C atoms, starting from a single S atom, in one sR1 cycle, the number of dubiously accepted atoms is 1, and the resulting model has 31 atoms:

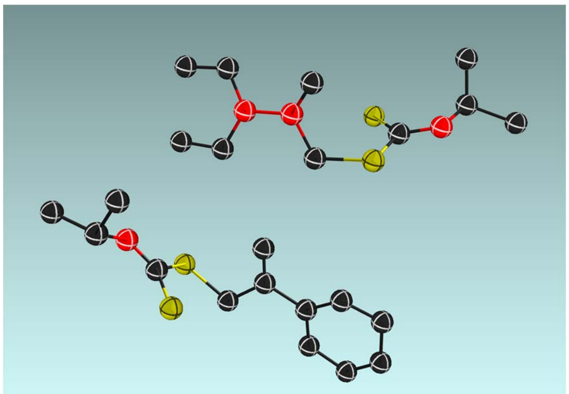

After another sR1 cycle, the resulting model has 32 atoms:

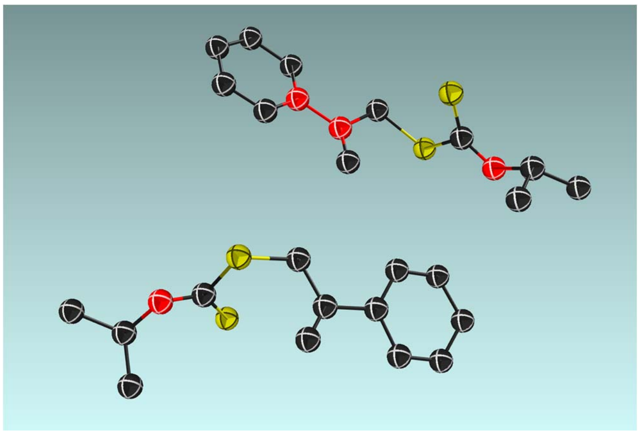

This matches the correct model.

Conclusion: In this case, padding 32 C atoms hurts the performance of the sR1 method slightly. However, the method is still working correctly after padding C atoms.
